# Analysis of factors associated with maintenance discontinuation in implant patients

**DOI:** 10.1186/s40064-015-1569-3

**Published:** 2015-12-12

**Authors:** Korenori Arai, Yoshihiro Takeda, Yurie Mori, Rie Terauchi, Takashi Furumori, Sachiko Tanaka, Tatsuro Miyake, Shunsuke Baba, Takayoshi Kawazoe

**Affiliations:** Department of Oral Implantology, Osaka Dental University, Hirakata, Japan; Department of biotatistics, Shiga University of Medical Science, Otsu, Japan; Department of Preventive and Community Dentistry, Osaka Dental University, Hirakata, Japan

**Keywords:** Implant treatment followed, Elderly patients, Health awareness, Behavior modification, After implant therapy, Dental implants

## Abstract

Maintenance following implant treatment is essential to ensure long-term stability. Accordingly, the objective of this study was to investigate the factors leading patients to discontinue maintenance following implant treatment. Among the 729 patients that underwent implantation at the Department of Oral Implantology, Osaka Dental University Hospital from January 2008 to December 2012, 41 patients were excluded from the study. Exclusion criteria comprised patients without a superstructure attachment, those who only underwent maxillary sinus floor augmentation procedures and those who discontinued visiting the hospital prior to superstructure attachment. Treatment was discontinued in 181 patients. The rate of discontinuation was 26.6 %. The odds ratio (OR) in the adjustment model was 1.552 (95 % CI 1.078–2.236) in males when compared with females. When compared with those who were 30–64 years old, the OR was 5.818 (95 % CI 3.017–11.220) in those 29 years old or younger and 1.561 (95 % CI 1.021–2.386) in those 65 years old or older. Moreover, when compared with those with a O’Leary’s Plaque Control Record of all teeth and superstructures (PCR) level of 20 % or less following superstructure attachment, the OR was 2.113 (95 % CI 1.471–3.035) in those with a PCR level of 20 % or more following superstructure attachment. It is highly important to decrease maintenance discontinuation, especially in patients aged 29 years old or younger with a PCR level of 20 % or more following superstructure attachment. Moreover, a support system must be developed to enable patients with difficulties visiting the hospital to continue their maintenance program.

## Background

In the clinical setting, prevention is becoming the focus of attention rather than therapy, along with changes in the understanding of dental disease. Considering that the prevalence of periodontal disease in adults is very high, the development of a preventive management system that periodically and efficiently controls the onset and progression of the disease, leading to a reduction in tooth loss, is essential (Hideto et al. 1998). In recent years, implant therapy has rapidly spread in Japan as a treatment method for the replacement of missing teeth, with good clinical performance having been achieved (Krebs et al. 2015; Karoussis et al. 2004; Haas et al. 1996; Buser et al. 2012; Lekholm et al. 1999; Covani et al. 2012; Lambrecht et al. 2003). Moreover, the survival rate of implants is lower in patients who do not undergo maintenance following therapy, and it has been found that these patients also have a higher incidence of prosthetic complications when compared with patients undergoing regular maintenance (Roccuzzo et al. 2010; Costa et al. 2012; Hultin et al. 2007). Therefore, continued maintenance is necessary to achieve long-term implant stability. However, there are a considerable number of patients who discontinue hospital visits for implant maintenance. Therefore, the objective of this study was to identify the factors associated with discontinuation of the maintenance phase in implant-treated patients.

## Patients and methods

### Research design

A case–controlled study design was employed, using the database of the Department of Oral Implantology, Osaka Dental University.

### Setting and participants

The participants consisted of 729 patients who underwent implant placement at the Department of Oral Implantology, Osaka Dental University Hospital from January 2008 to December 2012. The study was carried out until October 31, 2014.

### Exclusion criteria

Among the 729 patients who underwent implantation, 41 patients were excluded from the study based on the following criteria: patients without a superstructure attachment, patients who only underwent the maxillary sinus floor augmentation procedure, and patients who discontinued visiting the hospital prior to superstructure attachment.

### Investigation items

The following survey items were extracted for each participant: address, sex, age at implant placement, O’Leary’s Plaque Control Record of all teeth and superstructures (PCR) level at the initial visit, PCR level following attachment of the superstructure, the number of implants, and the number of missing teeth.

### Definition of the maintenance continuation and discontinuation groups

We defined patients who were continuing maintenance as of October 31, 2014 as the maintenance continuation group (herein after referred to as the continuation group), and patients who discontinued maintenance for 6 months or more as the maintenance discontinuation group (herein after referred to as the discontinuation group). Patients who discontinued maintenance a single time were placed in the discontinuation group, even if they later revisited the hospital for further maintenance.

### Variables

The aforementioned survey items collected from subjects in the continuation and discontinuation groups were set according to the following category variables. The patients were divided into two groups according to their address, i.e., whether it was within Osaka City (near the hospital location), or outside Osaka City. The age at implantation was divided into the three groups of ≤29 years old, 30–64 years old, and ≥65 years old. The PCR level at the initial visit was divided into two groups, ≤20 and >20 %, and the PCR level following attachment of the superstructure was divided into two groups, ≤20 and >20 %. Moreover, the number of implants and missing teeth in the continuation and discontinuation groups were used as continuous quantitative data.

### Data measurement method

We used surgery data from Osaka Dental University and the patients’ medical records to acquire relevant information. Participant groups were extracted from the surgery data book, with the participants’ survey items sampled from the medical records, and confirmation was made regarding whether maintenance was continued as of October 31, 2014.

### Statistical analysis

Among the 688 patients included in this study, 181 patients were in the discontinuation group, indicating a discontinuation rate of 26.6 %.

The χ^2^ test, Fisher’s exact test, and Mann–Whitney *U* test were conducted on each survey item in the continuation and discontinuation groups. Subsequently, binomial logistic regression analysis was carried out with discontinuation of maintenance as the dependent variable and patient address, sex, age at implant placement, number of implants, PCR at initial consultation, and PCR following attachment of the superstructure as the independent variables. The stepwise variable selection method by likelihood-ratio testing was used to select variables to measure the odds ratio (OR) with a 95 % confidence interval (CI). All survey items were used as category variables, while the number of implants and missing teeth were used as continuous quantitative data. The IBM SPSS Statistics 22 software program (IBM, Armonk, NY, USA) was used for statistical analysis.

### Ethical considerations

This study was implemented according to the modified declaration at Edinburgh, UK based on the Declaration of Helsinki (October 2000). A research plan taking into consideration the protection of human rights and benefits to the subjects was proposed, and the study was carried out upon approval from the Ethics Committee of Osaka Dental University (authorization number: Osaka Dental University Medical Ethics No. 110733) while ensuring the protection of personal information.

## Results

Table 1 provides a summary of the patients’ backgrounds. Although no statistically significant difference was observed between the continuation and discontinuation groups regarding patient addresses, the number of implants, the number of missing teeth, and the PCR level at the initial visit, a statistically significant difference was observed between the two groups regarding sex, age, and PCR level following attachment of the superstructure (Table 1).

**Table 1:** Characteristics of patients’ backgrounds at Osaka Dental University Hospital

	Continuation group	Discontinuation group	p value
Address			
Within Osaka city	180	73	
Outside Osaka city	325	110	0.326
Sex			
Female	326	91	
Male	179	92	0.001
Age			
30–64	389	106	
≤29	16	31	
65≤	100	46	<0.001
PCR^*^1			
20 % or less	143	38	
20 % or more	362	145	0.050
PCR^*^2			
20 % or less	306	70	
20 % or more	199	113	<0.001
Number of implants			
Median	2	2	0.141
Number of missing teeth			
Median	4	5	0.481

Proportions across levels of categorical variables were compared using the χ^2^ test and Fisher exact test. Medians for continuous variables were compared using the Mann–Whitney U test

*Age* age at implant placement, *PCR* ^*^ *1* PCR level at the initial visit, *PCR* ^*^ *2* PCR level following attachment of the superstructure

According to the results of a logistic regression analysis, sex, age, and PCR level following attachment of the superstructure were extracted as independent factors. At step 1 of the adjustment model, age was added as a variable; subsequently, PCR level following attachment of the superstructure was added as a variable at step 2, and sex was added as a variable at step 3. The OR of the adjustment model at step 3 was 1.6 (95 % CI 1.1–2.2) for males when compared with females. It was 5.8 (95 % CI 3.0–11.2) for those ≤29 years old in comparison with those 30–64 years old, and 1.6 (95 % CI 1.0–2.4) for those ≥65 years old. Moreover, it was 2.1 (95 % CI 1.5–3.0) for PCR level of >20 % when compared with ≤20 % following attachment of the superstructure (Table 2). The predictive value of differentiation between the predicted and actual value was 75.7 %.

**Table 2:** Adjusted odds ratios for maintenance discontinuation by case definition

	Odds ratio (95 % CI), p value		
	Step 1	Step 2	Step 3
Age			
30–64	1.0(ref)	1.0(ref)	1.0(ref)
≤29	7.1 (3.75–13.5), <0.001	6.0 (3.1–11.5), <0.001	5.8 (3.0–11.2), <0.001
65≤	1.7 (1.1–2.5), 0.012	1.7 (1.1–2.6), 0.013	1.6 (1.0–2.4), 0.040
PCR			
20 % or less		1.0(ref)	1.0(ref)
20 % or more		2.2 (1.6–3.2), <0.001	2.1 (1.5–3.0), <0.001
Sex			
Female			1.0(ref)
Male			1.6 (1.1–2.2), 0.018

These multivariate odds ratios for maintenance discontinuation were adjusted for address, sex, age at implant placement, *PCR* level at the initial visit, *PCR* level following attachment of the superstructure, the number of implants, and the number of missing teeth

*Age* age at implant placement, *PCR* PCR level following attachment of the superstructure

## Discussion and conclusion

In our department, the maximum maintenance interval following implant therapy is 6 months. Therefore, in this study, the discontinuation group was differentiated at 6 months.

Standards were set for each survey item and then stratified. A PCR value of ≤20 % was the target prior to implantation, so regarding the PCR level at the initial visit and following attachment of the superstructure, the subjects were divided into two groups, with 20 % as the standard. The subjects were divided into three groups, according to age, with the first group at ≤29 years old because the average age at first marriage in Japan is approximately 30 years old in both males and females. The third group, ≥65 years old, was created because the World Health Organization defines people aged 65 years or older as “elderly”. Regarding the patient addresses, the subjects were divided into two groups, those living inside and outside Osaka City, because the hospital associated with this study is located in Osaka City.

Revisits by the discontinuation group were not investigated in this study because it was difficult to conduct a follow-up on the reasons for such visits. Moreover, because the subjects were limited to patients visiting the university hospital, there was a lack of generalizability.

Therefore, these factors are considered to be limitations associated with this study. Because there are few objective investigations following implant therapy in other epidemiologic studies, providing evidence using the databases of multiple institutes may therefore be necessary in future studies.

The subjects of this study were not a representation of the general citizens of Japan, but rather consisted of people who underwent implant therapy at a university hospital, which is an expensive private medical expense and is not covered by the National Health Insurance System. In an awareness survey conducted following attachment of the superstructure, 41.7 % of patients (25/60) replied that they feel anxiety regarding cleaning the implant (Tomoko et al. 2007). Therefore, it is believed that the subjects of this study have a relatively high interest in oral hygiene when compared with the general public, and a greater understanding of the importance of maintenance. Periodical maintenance has a profound effect on preventing inflammation in the tissues surrounding both implants and natural teeth (Krebs et al. 2015; Karoussis et al. 2004; Haas et al. 1996; Buser et al. 2012; Lekholm et al. 1999; Covani et al. 2012; Lambrecht et al. 2003). Moreover, the lack of adhesion to supporting periodontal therapy (SPT) was associated with a higher incidence of bone loss and implant loss (Tsuyoshi et al. 2011; Chinatsu and Junko 2007) mentioned that in their maintenance discontinuation group, no significant difference was observed across sex, age, address, and the number of remaining teeth upon periodontal therapy (Kyoko et al. 1985; Mizuki et al. 1995, 2011). Similarly in our study, no significant difference was observed between the discontinuation and continuation groups regarding patient address, number of missing teeth, and number of implants; however, a significant difference was observed between the two groups regarding sex, age, and the PCR level following attachment of the superstructure. Moreover, the high discontinuation rate in patients aged ≤29 years old may be attributed to the reduced attention to health issues that is often observed among people in their twenties (2011).

There may have been a higher discontinuation rate in men compared with women because of difficulty visiting the hospital during consultation hours (9 am–5 pm on weekdays) because of their jobs. Accordingly, the development of a connection between our hospital and dental clinics allowing patients to visit outside office hours is necessary.

There have been reports that the number of patients visiting dental clinics greatly declines in patients aged 65 years or older (Roccuzzo et al. 2012; Frisch et al. 2014). 65 years old or more may have a time to spare regardless of sex. It is guessed that some discontinuation group patients aged ≥65 years became unable to make visits to our department because of old age. A new system must therefore be developed to allow the delivery of a maintenance program to patients who have difficulty making regular hospital visits because of old age.

Moreover, we must further emphasize the importance of maintenance following implant therapy in patients with a PCR level of >20 % following attachment of the superstructure. It is essential to motivate patients and help them to understand the importance of maintenance in association with implant therapy.

## Abbreviation

PCR: O’Leary’s plaque control record
