# Erratum to: Analysis of factors associated with maintenance discontinuation in implant patients

**DOI:** 10.1186/s40064-015-1637-8

**Published:** 2016-01-13

**Authors:** Korenori Arai, Yoshihiro Takeda, Yurie Mori, Rie Terauchi, Takashi Furumori, Sachiko Tanaka, Tatsuro Miyake, Shunsuke Baba, Takayoshi Kawazoe

**Affiliations:** Department of Oral Implantology, Osaka Dental University, Hirakata, Japan; Department of Biotatistics, Shiga University of Medical Science, Otsu, Japan; Department of Preventive and Community Dentistry, Osaka Dental University, Hirakata, Japan

## Erratum to: SpringerPlus (2015) 4:767 DOI 10.1186/s40064-015-1569-3

In the original article (Arai et al. 2015), there was an error in the way the author names were displayed. This has now been corrected in the published article and the publisher apologises for any inconvenience caused.
